# Cas rare de rupture bilatérale des tendons d'Achille sans notion de tendinopathie ou de chirurgie de la cheville chez un jeune sportif: à propos d'un cas et revue de la literature

**DOI:** 10.11604/pamj.2015.20.223.5985

**Published:** 2015-03-12

**Authors:** Amine Belmoubarik, Merouane Abouchane, Mohamed Amine Mahraoui

**Affiliations:** 1 Centre Hospitalier Universitaire Ibn Rochd, Casablanca, Maroc

**Keywords:** Tendon d′achille, rupture bilatérale, réparation, ciel ouvert, Achilles tendon, bilateral rupture, reparation, open surgery

## Abstract

Les lésions du tendon d'Achille sont plus fréquentes que celles du tendon quadricipital ou patellaire. Les lésions bilatérales sont par contre plus rares et sont souvent associées à une notion de tendinopathie, d'injection de corticoïdes ou de maladies systémiques tels que le lupus érythémateux, l'ostéomalacie ou l'insuffisance rénale chronique. Nous rapportons le cas d'un patient de 34 ans victime d'une rupture bilatérale des tendons d'Achille suite à une réception de saut lors de la pratique de gymnastique. Le patient n’était pas suivi pour maladie de système ou mis sous corticothérapie ou fluoroquinolones. Le diagnostic a été suspecté devant une dépression anormalement creusant le relief sous cutané des deux tendons d'Achille avec une impossibilité de flexion dorsale des deux chevilles en association à un signe de Thompson bilatéralement positif. L’échographie a confirmé le diagnostic. Le patient a été traité par laçage et suture tendineuse à ciel ouvert. La rupture bilatérale du tendon d'Achille est rare. La plupart des patients rapportent une notion de maladie systémique ou un antécédent de chirurgie du genou. Nous rapportons le cas d'une lésion rare dans la littérature, une rupture bilatérale des tendons achiléens sans notions de maladies auto-immunes ni de traitement aux corticoïdes. Les lésions bilatérales présentent certaines particularités thérapeutiques et évolutives. En effet, deux difficultés sont à noter la première réside dans l'absence de référence comparative pour régler et apprécier l’équin physiologique lors de la réparation tendineuse. La deuxième difficulté est l'obligation de différer l'appui à 45 jours. Ce qui est contraignant pour le patient. La technique de réparation à ciel ouvert couplée à une immobilisation en équin protégeant la suture nous a donné des résultats satisfaisants.

## Introduction

Les lésions du tendon d'Achille sont moins plus fréquentes que celles du tendon quadricipital ou patellaire. Ils surviennent le plus souvent chez un adulte jeune dont l’âge est inférieur à 40 ans suite à un traumatisme direct sur la cheville en hyperflexion dorsale ou en extension contrariée. Le tableau clinique est évident composé d'une douleur associée à une impotence fonctionnelle avec à l'examen une dépression à la palpation du tendon d'Achille, un signe de Thompson positif et une flexion dorsale active impossible. Les lésions bilatérales des tendons d'Achille sont extrêmement rares et sont souvent associées à une notion de tendinopathie, d'injection de corticoïdes ou de maladies systémiques tels que le lupus érythémateux, l'ostéomalacie ou l'insuffisance rénale chronique. Nous rapportons dans cet article le cas d'une rupture bilatérale des tendons d'Achille chez un patient jeune sans antécédents de tendinopathie ni de maladies systémiques.

## Patient et observation

**Figure 1:**
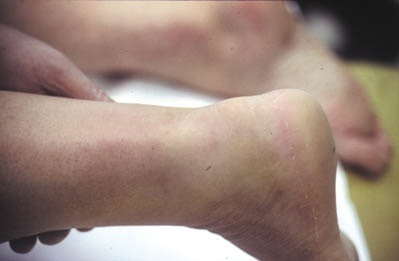
Encoche sur le trajet du tendon d'Achille

**Figure 2:**
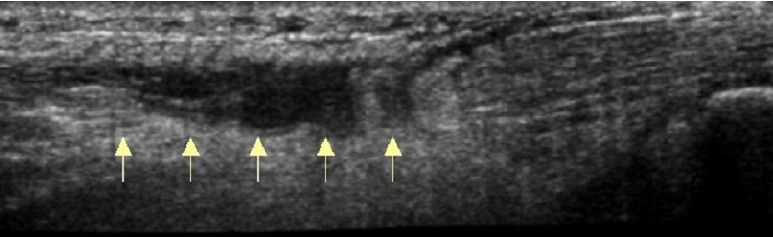
Échographie montrant une rupture du tiers moyen du tendon d'Achille en coupe sagittale

**Figure 3:**
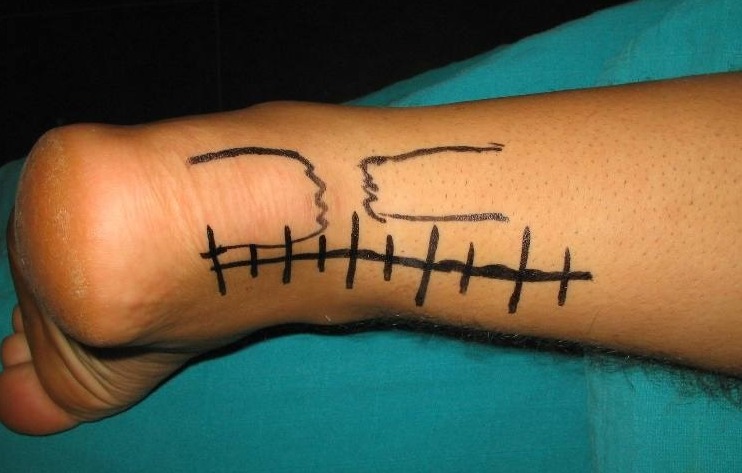
Photo montrant l'incision cutanée latéro-Achilléenne interne et la perte de substance tendineuse

Il s'agit d'un patient de 34 ans sans antécédents notables se présentant aux urgences dans un tableau de douleur intense au niveau des 2 chevilles avec impossibilité de la marche suite à un saut au gymnasium avec réception sur les deux pieds. L'examen clinique a objectivé une dépression au niveau du relief sous cutané des deux tendons d'Achille, siégeant pratiquement au même niveau à 2,5 cm de l'enthèse achilléenne. Le patient présentait également des difficultés de flexion dorsale active des deux chevilles. Les radiographies réalisées aux urgences objectivent comblement bilatéral des deux triangles Kager sans fracture associée. Une échographie a été également réalisée et qui a confirmé le diagnostic de rupture bilatérale des deux tendons achilléens (Figure 1, Figure 2, Figure 3). Le patient a été traité chirurgicalement le lendemain de son admission, sous rachianesthésie. Il a été installé en décubitus ventral avec un garrot pneumatique à la racine de chaque membre. Nous avons pratiqué une incision cutanée verticale postérieure parachilléenne médiale. Après incision verticale de la gaine tendineuse sur sa portion non lésée, le tendon a été libéré sur toute sa longueur et toutes ses faces. Les extrémités du tendon rompu ont été rapprochées et suturées avec renforcement par le grêle plantaire [1]. Pour protéger la suture, une mise en équin sous botte a été réalisée et gardée pendant 45 jours sans appui, relayée par une deuxième botte de marche réglable en position de flexion à 90^°^ pendant 45 jours avec autorisation d'appui. Le réglage de l’équin physiologique a été apprécié sur le tendon d'Achille où la rupture était la moins délabrante. L'appui a été différé à J45 vu que la lésion est bilatérale. La rééducation fonctionnelle a débuté au 45^ème^ jour post opératoire en favorisant la flexion-extension passive, avec renforcement de la chaîne des jumeaux et du soléaire, avec rééducation proprioceptive pendant la seconde échéance de 45 jours. Le patient a été revu régulièrement avec contrôle clinique et radiographique. Après un recul de 6 mois nous avons évalué l’évolution des 2 sutures en se basant sur l’étude de deux éléments: l'amplitude articulaire et la force de flexion dorsale de la cheville. Selon ces critères l’évolution clinique de notre patient a été jugée bonne pour la cheville gauche alors qu'elle est moyenne pour la cheville droite, côté où la lésion était délabrante.

## Discussion

La rupture du tendon d'Achille est la première lésion en terme de fréquence après la rupture du tendon quadricipital et la rupture du tendon patellaire qui survient en population sportive [1]. La lésion la plus fréquente est l'avulsion calcanéenne suivie de la rupture en plein corps [2] alors que le mécanisme le plus fréquent est la contraction violente ou contrariée du mollet (réception de saut, relèvement brusque d'une position accroupie) [3]. La rupture bilatérale du tendon d'Achille est rare. La plupart des patients rapportent une notion de maladie systémique ou un antécédent de chirurgie du tendon d'Achille [4]. Des signes inflammatoires peuvent être notés sur le siège de rupture en cas de lupus érythémateux [5] ou des dépôts amyloïdes en cas d'insuffisance rénale chronique [6]. Taylor et Lepilahti [7, 8] a réparti les ruptures des tendineuses selon leurs causes physiopathologiques en trois groupes: le premier est constitué de ruptures dues à des affections auto-immunes ou systémiques qui provoquent des changements de la structure du tendon. Le deuxième groupe est composé de ruptures dues à la prise de corticostéroïdes par voie orale ou par voie injectable. Le troisième groupe est fait de ruptures occasionnées par des microtraumatismes répétés. Le cas présenté dans cet article fait partie du troisième groupe, le patient n'a jamais été traité par des corticostéroïdes et n'a pas été pris en charge pour une maladie auto-immune ou de systèmes [9, 10]. Un bilan de maladies de système n'a pas été réalisé comme l'a préconisé Caldas pour tous les patients dont l’âge est supérieur à 40 ans vu que le patient est jeune [11–13]. Les ruptures bilatérales du tendon d'Achille présentent certaines difficultés thérapeutiques et évolutives. En effet l'absence de référence [14–16] comparative pour régler et apprécier l’équin physiologique lors de la réparation tendineuse vu qu'il est essentiel de placer les deux chevilles aux mêmes niveaux d'une part, et d'une autre part, on se retrouve devant l'obligation de différer l'appui à 45 jours, chose qui est manifestement contraignante [17] aussi bien pour le patient que pour son chirurgien. Des examens radiologiques tels que l’échographie et l'IRM [18–20] peuvent s'avérer utiles pour la détection de la rupture tendineuse en cas de doute diagnostic. La technique réparation tendineuse à ciel ouvert couplée à un renforcement par la plantaire grêle [21, 22] protégeant la suture avec immobilisation par botte nous a donné des résultats satisfaisants.

## Conclusion

Nous avons rapporté le cas d'une lésion rare dans la littérature, une rupture bilatérale des tendons d'Achille sans notion de maladies auto-immunes ni de traitement aux corticoïdes. Les lésions bilatérales présentent certaines particularités thérapeutiques et évolutives. Le renforcement par tendon de voisinage pour protéger la suture est essentiel dans les lésions de nature dégénératives.

## References

[1] Cetti R (1993). Operative versus nonoperative treatment of Achilles tendon rupture: a prospective randomised study and review of the literature. Am J Sports Med..

[2] Lecestre P, Germonville Th, Delplace J (1997). Ruptures du tendon d'Achille traitées par Ténorraphie percutanée: étude multicentrique de 61 cas, Annales orthop de l. Ouest..

[3] Fitzgibbons RE, Hefferon J, Hill J (1993). Percutaneous Achilles tendon repair. Am J Sports Med..

[4] Merti P, Jarde O, Tran Van F, Doutrellot P (1999). Tenorraphie percutanée pour rupture du tendon d'Achille: étude de 29 cas. Rev Chir Orthop..

[5] Kouvalchouk JF, Moujtahid M (1999). Réflexion à propos du traitement des ruptures du tendon d'Achille par suture percutanée. J Traumatol Sport..

[6] Häggmark T, Eriksson E (1979). Hypotrophy of the soleus muscle in man after Achilles tendon rupture: Discussion of findings obtained by computed tomography and morphologic studies. Am J Sports Med..

[7] Leppilahti J (1998). Outcome and prognostic factors of Achilles rupture repair using a new scoring method. Clin Orthop Relat Res..

[8] Ozkaya U, Parmaksizoglu AS, Kabukcuoglu Y, Sokucu S, Basilgan S (2009). Open minimally invasive Achilles tendon repair with early rehabilitation: Functional results of 25 consecutive patients. Injury..

[9] Cottalorda J, Kelberine F, Curvale G, Groulier P (1992). Traitement chirurgicale des ruptures du TA chez le sportif. J Chir Paris..

[10] Delponte P, Potier L, de Poulpiquet P, Buisson P (1992). Traitement des ruptures sous-cutanées du tendon d'Achille. Rev Chir Orthop..

[11] Khan RJK, Fick D, Keogh A, Crawford J, Brammar T, Parker M (2005). Treatment of acute Achilles tendon ruptures: a meta-analysis of randomized, controlled trials. JBJS..

[12] Crolla RM, van Leeuwen DM, van Ramshorst B, van der Werken C (1987). Acute rupture of the tendo calcaneus; surgical repair with functional after treatment. Acta Orthop Belg..

[13] Rouvillain JL, Navarre T, Labrada-Blanco O, Garron E, Daoud W (2008). Suture percutanée des ruptures fraîches du tendon calcanéen: à propos de 60 cas. J Traumatol Sport..

[14] Baulande G (1974). Ruptures sous cutanées du TA chez le sportif.

[15] Kouvalchouk JF, Rodineau J, Watin Augouard LW (1981). Les ruptures du TA: Comparaison des résultats du traitement opératoire et nom opératoire. RV ch orh..

[16] Lazrak KH, Tanane M, Guauia F, Bousselmane N (1998). Traitement chirurgical des ruptures du tendon d'achille à propos de 25 cas. Revue Marocaine de chirurgie orthopédique et traumatologique.

[17] Strauss EJ, Ishak C, Jazrawi L, Sherman O, Rosen J (2007). Operative treatment of acute Achilles tendon ruptures: an institutional review of clinical. Injury..

[18] Mc Comis G, Nawoczenski DA, Dehaven KE (1997). Functional bracing for rupture of the Achilles tendon. J Bone Joint Surg..

[19] Laffenetre O, Cermolancce C, Coillard JY, De Lavigne C, Determe P, Diebold P (2004). Ténolig et sport: étude prospective d'une série de 35 patients évalués par étude isocinétique et revue à un an de recul.

[20] Khan RJ, Carey Smith RL (2010). Surgical interventions for treating a cute Achilles tendon ruptures. Cochrane Database Syst Rev..

[21] Carter TR, Fowler PJ, Blokker C (1992). Functional postoperative treatment of Achilles tendon repair. Am J Sports Med..

[22] Cottalorda J, Kelberine F, Curvale G, Groulier P (1992). Traitement chirurgicale des ruptures du TA chez le sportif. J Chir Paris..

